# Notch1 binds and induces degradation of Snail in hepatocellular carcinoma

**DOI:** 10.1186/1741-7007-9-83

**Published:** 2011-11-30

**Authors:** Seung-Oe Lim, Hyeon Seop Kim, Xiaoyuan Quan, Sun-Min Ahn, Hongtae Kim, David Hsieh, Je Kyung Seong, Guhung Jung

**Affiliations:** 1 Department of Biological Sciences, Seoul National University, 1 Gwanak-ro, Gwanak-gu, Seoul, 151-747, Korea; 2 Department of Biological Science, Sungkyunkwan University, Seobu-ro, Jangan-gu, Suwon, Gyeonggi-do, 110-745, Korea; 3 Laboratory of Developmental Biology and Genomics, College of Veterinary Medicine, Seoul National University, 1 Gwanak-ro, Gwanak-gu, Seoul, 151-747, Korea

**Keywords:** Snail, Notch1 intracellular domain, degradation, invasion, hepatocellular carcinoma

## Abstract

**Background:**

Hepatocellular carcinoma (HCC) is a common, highly invasive malignant tumor associated with a high mortality rate. We previously reported that the aberrant expression of Snail via activation of reactive oxygen species contributes to the invasive property of HCC, in part by downregulation of E-cadherin through both transcriptional repression and epigenetic modification of the E-cadherin promoter. Having demonstrated the ability of Snail to bind and recruit histone deacetylase 1 and DNA methyltransferase 1 in this context, we set out to look for other interactions that could affect its ability to promote oncogenic transformation and cancer cell invasion.

**Results:**

Using cells that stably expressed Snail, we characterized Snail protein interactors by tandem affinity purification and mass spectrometry. Immunoprecipitation and subcellular colocalization studies were performed to confirm our identification of the Notch1 intracellular domain (NICD) as a novel Snail-binding partner. NICD interaction with Snail was found to induce ubiquitination and MDM2-dependent degradation of Snail. Interestingly, NICD inhibited Snail-dependent invasive properties in both HCC cells and mouse embryonic fibroblasts.

**Conclusions:**

Our study demonstrates that NICD can oppose Snail-dependent HCC cell invasion by binding and inducing proteolytic degradation of Snail. Although Notch signaling and Snail are both widely considered tumor-promoting factors, our findings indicate that the individual oncogenic contribution of Notch1 and Snail in malignant systems should be interpreted carefully, particularly when they are conjointly expressed.

## Background

Hepatocellular carcinoma (HCC) is a common, highly invasive malignant tumor associated with a high mortality rate [1,2]. The resistance of HCC to existing antineoplastic agents and the limited effectiveness of chemotherapies due to underlying liver disease contribute to the poor prognosis for patients with HCC [3]. Although surgical resection is the preferred standard of care for patients with HCC, few patients are suitable candidates for this treatment and recurrence is common even after radical curative resection [3,4]. Given the inadequate impact of conventional therapies and the rising incidence of HCC, elucidation of the oncogenic mechanisms underlying HCC development is critical for identifying potential therapeutic targets or modalities.

Snail is a well-known zinc finger (ZF) transcriptional repressor responsible for epithelial-to-mesenchymal transitions (EMTs) and metastasis in several cancers [5,6]. We previously reported that upregulation of human *SNAI1 *(Snail) expression by reactive oxygen species contributes to the invasive nature of HCC, in part by inducing the expression of matrix metalloproteinases and downregulating E-cadherin expression through both transcriptional repression and epigenetic modification of the E-cadherin promoter [7]. Other recent reports have revealed that Snail induces broad epigenetic modifications of target genes by interacting with tumor-associated proteins (including HDAC1, DNMT1 and p53) [7-9]. Taken together, the ability of Snail to promote oncogenic transformation and cancer cell invasion is likely mediated by its interactions with other proteins in addition to its transcriptional activity.

The Notch signaling pathway regulates embryonic cell determination and differentiation as well as postnatal development [10,11]. Although alterations in the Notch pathway are associated with malignant processes, there is also evidence that supports a tumor-suppressive role for Notch signals [12]. Activation of the Notch pathway is initiated through juxtacrine ligand-receptor interactions and the proteolytic cleavage of Notch1 by γ-secretase, which liberates the *Notch1 *intracellular domain (NICD) from the membrane, allowing NICD to translocate to the nucleus [10]. Nuclear NICD then associates with CSL (CBF1/RBPJk in vertebrates, Suppressor of hairless in *Drosophila*, Lag-1 in *Caenorhabditis elegans*) transcriptional factors to inhibit CSL transcriptional repression of Notch target genes [10]. Of note, though both NICD and Snail proteins are known to play a central role in cancer cell growth, invasion and metastasis, Notch signaling is also capable of inhibiting HCC tumor growth through the induction of cell-cycle arrest and apoptosis [5,13-15].

In this study, we have identified NICD as a novel Snail binding partner by using tandem affinity purification and mass spectrometry (MS/MS) in HCC cells. Using HCC cell lines and mouse embryonic fibroblasts (MEFs), we demonstrate that NICD can induce Snail degradation and impede Snail-dependent cell invasion. Although Snail is known to be degraded by the β-TrCP1 and FBXL14 E3 ubiquitin ligases, our data indicate that NICD-mediated Snail degradation may instead be dependent on MDM2 [16,17].

## Results

### NICD is a novel binding partner of Snail

**Figure 1:**
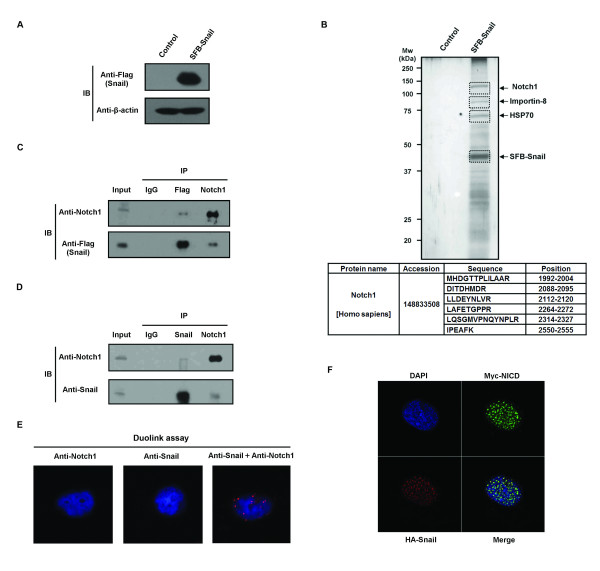
**Notch1 intracellular domain is a Snail binding partner**. **(A) **Expression of the S protein-Flag-SBP (streptavidin-binding peptide) (SFB)-Snail protein in stably transfected cell lines was assessed by immunoblot analysis. **(B) **Tandem affinity purification of Snail-bound protein complexes was conducted using 293T cells stably expressing SFB-tagged Snail. Bound proteins were separated by SDS-PAGE and examined by silver staining. The Notch1 protein and the number of peptides identified by mass spectrometry are shown in the table at the bottom. "Position" indicates the position of the peptide in the amino acid sequence of Notch1. **(C) **SFB-Snail-overexpressing cells were treated with 10 μM MG132 for 6 hours and coimmunoprecipitated using anti-Flag and anti-Notch1 antibodies. Immunoblot analysis showed an interaction between Flag-Snail and Notch1 intracellular domain (NICD). Immunoglobulin G (IgG) served as a negative control. **(D) **Hep3B cells were treated with 10 μM MG132 for 6 hours and coimmunoprecipitated using anti-Snail and anti-Notch1 antibodies. Immunoblot analysis showed an interaction between endogenous Snail and the Notch1 (NICD) protein. IgG served as a negative control. **(E) **Hep3B cells were immunostained with anti-Snail and/or anti-Notch1 antibodies and assessed using the Duolink II assay. Red foci indicate interactions between endogenous Snail and Notch1 proteins. **(F) **Hep3B cells were transfected with hemagglutinin (HA)-Snail and Myc-NICD and then stained with anti-HA and anti-Myc antibodies. Cellular localization of Snail (red) and NICD (green) was examined. Nuclei were stained with 4', 6-diamidino-2-phenylindole (DAPI blue).

To elucidate regulatory factors of Snail, we sought to identify its binding partners by performing MS/MS (Figures 1A and 1B and Additional file 1). We identified Notch1 as a Snail-associated protein (Figures 1B and 1C) and confirmed an endogenous Snail and Notch1 interaction by performing coimmunoprecipitation and immunoblot assays (Figure 1D). In both analyses, the size of the detected Notch1 protein was approximately between 100 and 150 kDa, suggesting that Snail binds to the proteolytically cleaved intracellular domain of Notch1, known as NICD. Using the Duolink II assay (Olink Bioscience, Uppsala, Sweden), we have shown that endogenous Snail and Notch1 proteins interact in multiple cancer lines (Figure 1E and Additional file 2, with each red dot in the images representing a fluorescent signal from a Snail-Notch1 interaction). Colocalization of NICD and Snail in nuclear foci by immunofluorescence corroborates the interaction of the two proteins (Figure 1F).

### The ANK domain of NICD and the zinc finger domain of Snail are required for NICD and Snail interaction

**Figure 2:**
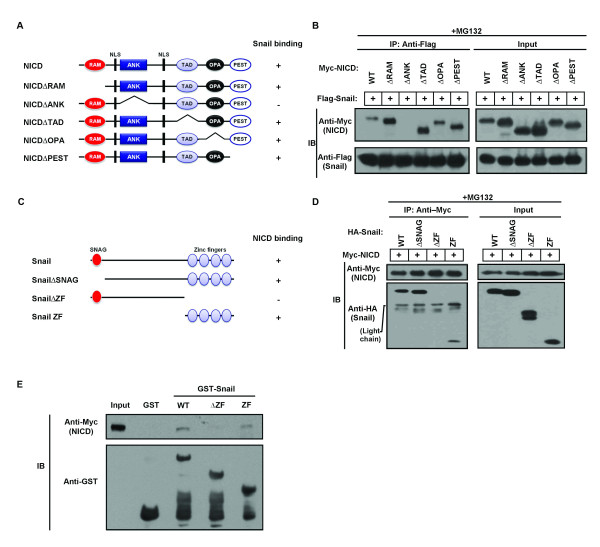
**Domain mapping of the Notch1 intracellular domain-Snail complex**. **(A) **Schematic of Notch1 intracellular domain (NICD) and deletion mutants. The ability of each fragment to bind Snail is indicated on the right. **(B) **293T cells were cotransfected with Flag-Snail and Myc-NICD deletion mutants. After transfection, cell lysates were immunoprecipitated with anti-Flag antibody and assessed by immunoblot analysis. The NICD ankyrin/cdc10 repeat (ANK) domain ΔANK mutant did not bind the Snail protein. NLS, nuclear localization signal. **(C) **Schematic of Snail and deletion mutants. The ability of each fragment to bind NICD is indicated on the right. **(D) **293T cells were cotransfected with Myc-NICD and Flag-Snail deletion mutants. After transfection, cell lysates were immunoprecipitated with anti-Myc antibody and assessed by immunoblot analysis. The Snail zinc finger (ZF) ΔZF mutant did not bind the NICD protein. **(E) **Glutathione *S*-transferase (GST)-Snail wild-type (WT), ΔZF and ZF mutants were incubated with cell lysates of Huh7 cells that overexpressed Myc-NICD. The bound proteins were eluted by boiling the cells in SDS sample buffer for 10 minutes and then were analyzed by immunoblotting with anti-Myc and anti-GST antibodies.

NICD contains a RBP-J κ-associated module (RAM) domain, seven ankyrin/cdc10 repeats (ANK) domain, two nuclear localization signals, a transcriptional transactivation domain (TAD), a polyglutamine tract (OPA) domain and a proline-, glutamic acid-, serine- and threonine-rich (PEST) domain [10] (Figure 2A). Snail contains a highly conserved C-terminal region, a ZF domain and a divergent N-terminal (SNAG) domain (Figure 2C) [5]. We mapped interacting domains of NICD and Snail by coimmunoprecipitation analysis using deletion mutants of both proteins and found that the Snail ZF domain and the NICD ANK domain were responsible for the interaction between the two molecules (Figures 2B and 2D). To exclude the possibility that altered cellular localization was responsible for the inability of the Snail-ΔZF mutant to bind NICD, we performed glutathione *S*-transferase (GST) pull-down assays using GST-Snail wild-type (WT), ΔZF and ZF mutants, which showed that only the Snail-ΔZF mutant did not interact with NICD (Figure 2E).

### NICD downregulates Snail protein

**Figure 3:**
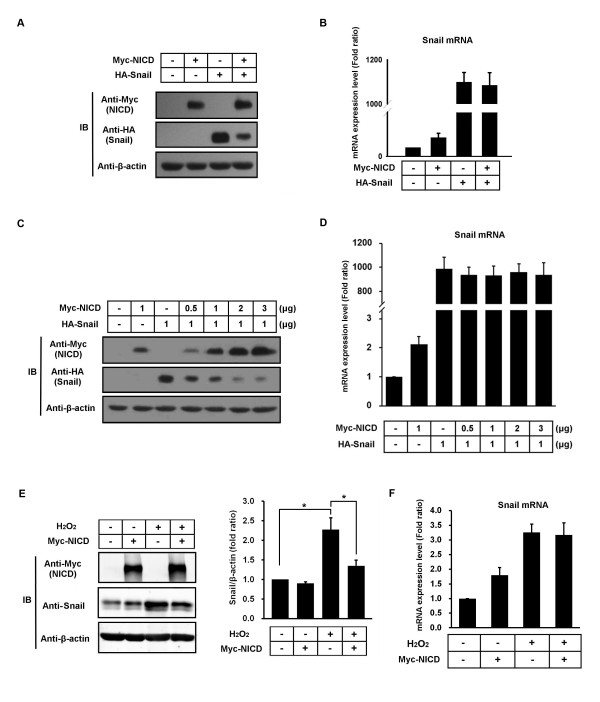
**Notch1 intracellular domain downregulates Snail protein**. **(A) **Hep3B cells were transfected with Myc-Notch1 intracellular domain (NICD) and/or hemagglutinin (HA)-Snail and evaluated for the expression of both proteins using immunoblot analysis. **(B) **Hep3B cells were transfected with Myc-NICD and/or HA-Snail and evaluated for the expression of Snail mRNA using RT-PCR. **(C) **Hep3B cells were transfected with HA-Snail and/or different doses of Myc-NICD, then evaluated for the expression of both proteins using immunoblot analysis. β-actin served as an internal control. **(D) **Hep3B cells were transfected with HA-Snail and/or different doses of Myc-NICD and evaluated for the expression of Snail mRNA using RT-PCR. β-actin served as an internal control. **(E) **Hep3B cells were transfected with Myc-NICD and/or treated with 300 μM H_2_O_2 _for 72 hours, then evaluated for the expression of NICD and Snail proteins using immunoblot analysis. Densitometry results of Snail analysis are shown in the right panel bar graph. **(F) **Hep3B cells were transfected with Myc-NICD and/or treated with 300 μM H_2_O_2 _for 72 hours, then evaluated for Snail mRNA expression using RT-PCR. β-actin served as an internal control. Bar graphs show the means ± SD of three independent experiments.

To clarify the role of the interaction between NICD and Snail, we transfected Hep3B cells with hemagglutinin (HA)-Snail and Myc-NICD constructs. Interestingly, we observed that the HA-Snail protein was dramatically decreased in a dose-dependent manner in the presence of Myc-NICD (Figures 3A and 3C). Under the same experimental conditions in which NICD and Snail were coexpressed, there was no change in the level of Snail mRNA in comparison with cells that expressed HA-Snail alone (Figures 3B and 3D). Furthermore, we performed additional experiments using endogenous Snail protein-upregulated conditions. In our previous study, reactive oxygen species (ROS) stress such as H_2_O_2 _treatment-upregulated Snail mRNA and protein expression [7]. In the H_2_O_2_-treated cells, Myc-NICD downregulated the Snail protein level, not the mRNA level, although in normal conditions Myc-NICD upregulated Snail mRNA expression (Figures 3E and 3F). Collectively, these results suggest that NICD may decrease Snail expression through a posttranscriptional mechanism.

### NICD induces degradation of the Snail protein by ubiquitination

**Figure 4:**
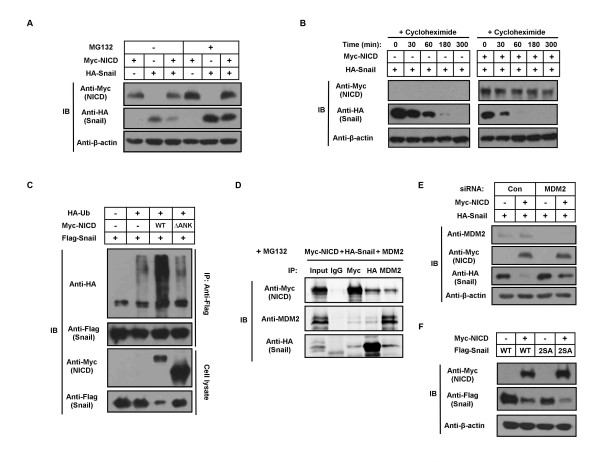
**Notch1 intracellular domain induces ubiquitination and degradation of the Snail protein**. **(A) **Hep3B cells were transfected with Myc-Notch1 intracellular domain (NICD) and/or hemagglutinin (HA)-Snail, then treated with MG132 for 6 hours. Expression of Myc-NICD and HA-Snail was then analyzed by immunoblotting. **(B) **HA-Snail- or HA-Snail/Myc-NICD-transfected Hep3B cells were treated with 20 μM cycloheximide for different time intervals, then evaluated for Snail protein levels using immunoblot analysis. **(C) **293T cells were transfected with Myc-NICD, SFB-Snail and/or HA-tagged ubiquitin, then treated with MG132 for 6 hours. Snail protein was immunoprecipitated using an anti-Flag antibody. Cell lysates were analyzed by immunoblotting with the indicated antibodies. **(D) **Hep3B cells were transfected with Myc-NICD, HA-Snail and MDM2; coimmunoprecipitated using anti-Myc and anti-MDM2 antibodies, respectively; and evaluated by immunoblot analysis. Immunoglobulin G served as a negative control. **(E) **Hep3B cells were transfected with Myc-NICD, HA-Snail and/or MDM2 siRNA and evaluated for the expression of Myc-NICD, HA-Snail and MDM2 using immunoblot analysis. **(F) **Hep3B cells were transfected with Myc-NICD and/or Flag-Snail wild-type (WT) or 2SA mutant. The expression of Myc-NICD and Flag-Snail was then analyzed by immunoblotting. β-actin was used as an internal control.

NICD-induced decrease of Snail protein was inhibited by treatment with the proteasome inhibitor MG132, suggesting that NICD promotes Snail degradation (Figure 4A). Treatment of Snail and NICD cotransfected cells with cycloheximide, an inhibitor of protein biosynthesis, revealed that the protein half-life of Snail is shortened in the presence of NICD (Figure 4B). As Snail is known to be tagged by ubiquitin for proteolytic degradation, we sought to determine whether NICD could induce Snail ubiquitination by examining ubiquitinated Snail in cells cotransfected with Flag-Snail and Myc-NICD WT or Myc-NICD ΔANK mutant (Mut). Because NICD ΔANK Mut does not interact with the Snail protein, we hypothesized that Snail would not be ubiquitinated in its presence. In agreement with this hypothesis, ubiquitinated Flag-Snail was detected only in Flag-Snail and Myc-NICD WT cotransfected cells and not in Flag-Snail and ΔANK Mut transfected cells (Figure 4C). These data support a role for NICD in regulating ubiquitin-dependent Snail degradation.

Coimmunoprecipitation experiments showed that Myc-NICD can interact with MDM2 (Figure 4D). In a previous report, we found that Snail may also bind MDM2 (Figure 4D) [18]. These data support the notion that NICD induces degradation of the Snail protein via MDM2-mediated ubiquitination because NICD has no known intrinsic E3 ligase function. Indeed, in Hep3B cells cotransfected with Myc-NICD and MDM2 siRNA, NICD-induced Snail degradation was decreased compared to control siRNA-transfected cells (Figure 4E). Glycogen synthase kinase 3β (GSK-3β) is known to induce Snail phosphorylation and degradation through the E3 ubiquitin ligase β-TrCP1 [17]. To determine whether NICD-induced Snail degradation is reliant on GSK-3β/β-TrCP1, we analyzed the protein level of the Snail 2SA mutant, which has two point mutations in the β-TrcP motif, in cells cotransfected with Snail 2SA mutant and NICD. We found that both the Snail WT and 2SA mutant proteins were uniformly degraded by NICD, implying that NICD-induced Snail degradation is a β-TrcP-independent phenomenon (Figure 4F).

### NICD induces degradation of endogenous Snail protein

**Figure 5:**
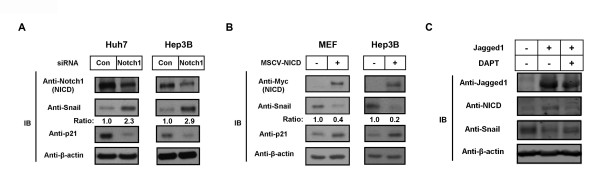
**Notch1 intracellular domain induces the degradation of the Snail protein at endogenous levels**. **(A) **Huh7 and Hep3B cells were transfected with control siRNA and Notch1 siRNA, then evaluated for the expression of Notch1 intracellular domain (NICD) and Snail using immunoblot analysis. Densitometry results of Snail expression are given as intensity ratios listed below the blot. Con, control. **(B) **Mouse embryonic fibroblast (MEFs) and Hep3B cells were infected with murine stem cell virus (MSCV) or MSCV-NICD. After puromycin selection, cells were treated with 300 μM H_2_O_2 _for 72 hours and then were evaluated for endogenous Snail protein levels using immunoblot analysis. **(C) **Huh7 cells were infected with MSCV-Jagged1, then treated with 300 μM H_2_O_2 _for 72 hours. The expression of Jagged1, NICD and Snail was then analyzed by immunoblotting. DAPT (*N*-[*N*-(3, 5-difluorophenacetyl)-L-alanyl]-*S*-phenylglycine *t*-butyl ester), an inhibitor of γ-secretase, was used to inhibit NICD activation. β-actin was used as an internal control.

To determine whether NICD could regulate endogenous levels of Snail protein, we treated Hep3B and Huh7 cells with Notch1 siRNA to silence NICD expression. In Notch1 siRNA-transfected cells, endogenous Snail protein levels were elevated relative to control siRNA-transfected cells (Figure 5A). We confirmed these results using a murine stem cell virus (MSCV) retroviral system to express NICD (MSCV-NICD) in MEFs and Hep3B cells. In both cell types, NICD expression led to decreased levels of endogenous Snail protein but upregulation of p21, an established Notch target gene (Figure 5B). Correspondingly, expression of Jagged1, an activating ligand of Notch signaling, repressed Snail protein levels only in the absence of DAPT (*N*-[*N*-(3, 5-difluorophenacetyl)-L-alanyl]-*S*-phenylglycine *t*-butyl ester), a γ-secretase inhibitor (Figure 5C). Taken together, our results thus far show that physiological activation of the Notch pathway or NICD expression is necessary and sufficient for Snail degradation.

### Snail and NICD regulate the invasiveness of HCC cells

**Figure 6:**
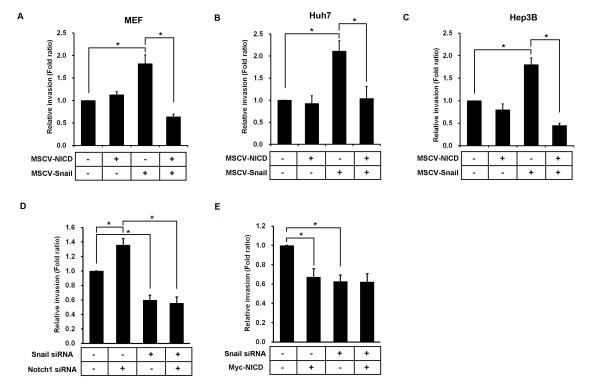
**Snail and Notch1 intracellular domain regulate invasion**. Mouse embryonic fibroblast (MEFs) **(A)**, Huh7 cells **(B) **and Hep3B cells **(C) **were infected with murine stem cell virus-Notch1 intracellular domain (MSCV-NICD) and/or MSCV-Snail, selected in puromycin, and assayed for cell invasion using the Oris Cell Invasion and Detection Kit. **(D) **Hep3B cells were transfected with Notch1 and/or Snail siRNA, then assayed for cell invasion. **(E) **Hep3B cells were transfected with Myc-NICD and/or Snail siRNA, treated with 300 μM H_2_O_2 _for 72 hours and assayed for cell invasion. Bar graphs show the means ± SD of three independent experiments. *P < 0.05 statistical significance of the three experiments was determined using Student's *t*-test.

To understand the function of the NICD-Snail interaction, we infected primary MEFs with the MSCV driving Myc-NICD and/or Flag-Snail genes and evaluated Snail protein levels. Predictably, in Myc-NICD and Flag-Snail coinfected samples, NICD-induced Snail degradation was observed (Additional file 3). Given that Notch1 and Snail signaling individually regulate cell invasion, we considered whether their interaction might perturb their native functions [5,13]. Using a cell invasion assay, we found that the number of invading Snail and NICD coinfected cells was significantly less than that of cells infected by Snail or NICD alone (Figure 6A). Parallel findings were observed in Huh7 and Hep3B cells in which coexpression of Snail and NICD suppressed invasiveness (Additional file 3 Figure 6B, and 6C). Additionally, the number of invading cells was much greater in cells that had been transfected with Notch1 siRNA than those transfected with control siRNA (Figure 6D). However, the number of invading cells transfected with both Notch1 and Snail siRNAs did not increase (Figure 6D).

To test whether NICD specifically inhibits Snail-induced cell invasion, we performed invasion assays in NICD and/or Snail siRNA-transfected Hep3B cells after treatment with H_2_O_2_. As portrayed in Figure 6E, we found that H_2_O_2_-treated cells ectopically expressing NICD were less invasive than H_2_O_2_-treated control cells, which correlates with the ability of NICD to inhibit H_2_O_2_-dependent upregulation of Snail expression (Figure 3E). However, in H_2_O_2_-treated cells cotransfected with Snail siRNA, NICD expression lacked any effect on cell invasion (Figure 6E and Additional file 3). Taken together, these results demonstrate that NICD can inhibit Snail-induced cell invasion.

## Discussion

Previously, Notch1 was shown to cooperate with the Snail pathway by upregulating Snail transcription, inducing EMT and promoting hypoxia-induced tumor cell invasion [19]. However, we found that NICD, which is an intracellular functional molecule liberated from Notch1, can suppress Snail protein via direct binding. This suggests that the Notch1-Snail pathway includes not only the single functional pathway but also a reciprocal interrelationship. Although both NICD and Snail protein play a central role in cancer cell growth, invasion and metastasis [5,13-15], their physical interaction has not been described previously. Given the highly invasive phenotype of HCC and the overexpression of Snail in HCC tissue [7], we utilized HCC cell lines as a model cell system in which to study the functional involvement of NICD-Snail interaction in disease development.

Notch1 is known to regulate Snail and Slug mRNA levels, but efforts have not been made to examine alternative functions of NICD and Snail expression in the same cancer cell line [19-21]. In this study, we have demonstrated the ability of NICD to associate with Snail to induce its ubiquitination and proteasomal degradation. NICD has been reported to upregulate Snail activity by inducing Snail mRNA expression under hypoxic conditions [19]. In addition, Notch1 is related to the mesenchymal program by activating Snail expression in cardiac development [22,23]. Our findings appear to be inconsistent with these previous results. However, because Notch signals and cellular functions vary according to cell type and cellular environment, these inconsistencies could be caused by the different cell types and conditions. Although ectopic expression of NICD slightly induced Snail transcription, we postulate that the genetic interaction between Snail and NICD and their physical association may be physiologically exclusive events, which may account for their contrasting cellular effects. In our previous study, ROS stress upregulated Snail mRNA and protein expression [7]. In the ROS-treated cells, Myc-NICD downregulated the Snail protein level, not the mRNA level (Figure 3). These latter data indicate that, under conditions of exogenous Snail expression or endogenous Snail upregulation by ROS stress, NICD reduces Snail protein levels by inducing Snail degradation without affecting Snail transcription. Because the NICD ΔANK mutant failed to induce ubiquitination and degradation of Snail, it appears that the physical interaction of NICD and Snail is required for the degenerative process. NICD is not known to have inherent E3 ligase functions, indicating that NICD may initiate or serve as a cofactor of the degradation signal. We have shown that Myc-NICD interacts with MDM2 (Figure 4D), but the NICD ΔANK mutant did not (data not shown). In addition, in MDM2 siRNA-transfected cells, NICD-induced Snail degradation was decreased compared to control siRNA-transfected cells (Figure 4E), suggesting that MDM2 has a role in NICD-induced Snail degradation.

We attempted to improve the transient transfection conditions with a stably NICD-expressing cell line based on a retroviral expression system. In these cells, the expression levels of NICD were much lower than those found with transient transfection. As shown in Figure 5 and Additional file 3 NICD-induced Snail degradation occurred in our stably NICD-expressing cells. In addition, to show that ligand-stimulated, NICD-induced Snail degradation took place, we used the cell line that expressed the exogenous Jagged1 ligand. In these cells, NICD induced Snail degradation (Figure 5C). Moreover, Notch1 siRNA upregulated endogenous Snail protein levels, as shown in Figure 5A. These data suggest that NICD-induced Snail degradation occurs in certain physiological conditions.

In this study, NICD-induced degradation of Snail inhibited Snail-dependent invasive behavior, as expected. Consistent with these invasion data, E-cadherin expression decreased in Snail-overexpressed cells, but it did not in Snail- and NICD-coexpressed cells compared to control cells (Additional file 3). In our previous study, we reported p53-induced Snail degradation via MDM2 [18]. Notch1 expression and signaling were regulated differently, depending on p53 status [13,24]. In other systems, Notch1 related to the mesenchymal program by activating Snail expression [19,22,23]. In another previous study, we showed that Notch1 and Snail differentially regulate invasion of HCC cells depending on p53 status [25]. The inhibition of invasion by NICD and Snail coexpression was observed in both Hep3B cells (p53-null) and Huh7 cells (p53-mutant). On the other hand, in p53 WT cells such as HepG2, NICD and Snail coexpression promotes invasiveness [25]. In the present study, we showed NICD regulated invasion via Snail degradation in p53-null or p53-mutant status. On the basis of these reports, we suspect that the interactions among p53, MDM2, Notch1 and Snail play an important role in EMT. Furthermore, these data explain the context-dependent regulation of EMT by the Notch signaling system. Our study provides one clue for understanding the complex regulation mechanism of p53, MDM2, Notch1 and Snail in the EMT process. The regulation of these proteins and their physiological contribution to HCC development and malignant behavior require further investigation. However, the mechanism that we have described presents substantial evidence of cross-interference between the Notch and Snail signaling pathways, which is mediated by their direct binding.

## Conclusions

Herein we have identified NICD as a novel binding partner of Snail. Our results indicate that the interaction between NICD and Snail promotes Snail ubiquitination and degradation through an MDM2-dependent mechanism. Accordingly, we have shown that NICD impairs Snail-associated cell invasion in a conserved manner between MEFs and HCC cells. These findings collectively suggest that in the instance in which Notch1 and Snail are conjointly present, Notch signaling may serve as an antagonist of Snail function. The results of our research ultimately support caution in the use of genetic or pharmacological strategies that solely target the Notch pathway for therapeutic treatment in Snail-associated HCC. Further investigation is warranted to fully determine the precise cellular processes disrupted by Notch inhibition and thus to better assess the therapeutic value and clinical implications of Notch pathway antagonists.

## Methods

### Cell culture and treatments

Hep3B human hepatoma cells, Huh7 human hepatoma cells, HT-29 human colorectal carcinoma cells, Panc-1 human pancreatic cancer cells, MDA-MB231 human breast carcinoma cells and 293T cells were cultured in DMEM with 10% fetal bovine serum (FBS). In some experiments, cells were incubated with 10 μM MG132 for 6 hours. To establish stable 293T lines expressing human triple-tagged Snail, we inserted the Snail cDNA sequence into the pIRES2-EGFP/SFB vector and transfected the 293T cells with either pIRES2-EGFP/SFB as a control or pIRES2-EGFP/SFB-Snail with FuGENE 6 transfection reagent (Roche Applied Science, Penzberg, Germany). G418 sulfate (Geneticin; Invitrogen, Carlsbad, CA, USA) or puromycin (InvivoGen, San Diego, CA, USA) were used to select colonies. Primary MEFs obtained from embryos of p53^-/- ^mice at day 13.5 were cultured in DMEM supplemented with 10% FBS. For siRNA experiments, cells were transfected with Notch1, Snail, MDM2 and control siRNA (ON-TARGET*plus *siRNA reagents; Dharmacon/Thermo Fisher Scientific, Lafayette, CO, USA) using Oligofectamine transfection reagent (Invitrogen/Life Technologies). After a 72-hour transfection or transduction, protein expression was analyzed by immunoblotting. All other reagents were purchased from Sigma-Aldrich (St Louis, MO, USA).

### Tandem affinity purification and mass spectrometry analysis

For purification, cells stably expressing Snail tagged with S protein-Flag-SBP (streptavidin-binding peptide) (SFB) were lysed with 50 mM Tris·HCl, pH 8.0, 150 mM NaCl, 5 mM ethylenediaminetetraacetic acid (EDTA) and 0.5% Nonidet P-40 (NP-40) (NTEN) lysis buffer supplemented with protease and phosphatase inhibitors. Streptavidin Sepharose (first purification) followed by S-beads (second purification) were used to bind SFB-tagged Snail. Snail complexes were reduced, alkylated and digested overnight with trypsin. Peptides were characterized using MS/MS (Thermo Fisher Scientific, Inc, Waltham, MA, USA). The resulting peptide tandem mass spectra were searched against a comprehensive nonredundant protein database using SEQUEST Cluster software (Thermo Fisher Scientific, Inc).

### Plasmids

Human *SNAI1 *and NICD were obtained from the Huh7 cell line and cloned into pCMV/HA, pCMV/Myc and/or p3 × Flag/CMV expression vectors. Using pCMV/HA-Snail as a template, a SNAG domain-deleted Snail construct (ΔSNAG), ZF region-deleted Snail construct (ΔZF) and ZF region construct were developed by performing PCR with the following primers: 5'-AGAATTCGGAAGCCCTCCGAC-3' (forward for ΔSNAG), 5'-AGCGGCCGCTCAGCGGGGACATCC-3' (reverse for ΔSNAG), 5'-AGAATTCCGCGCTCTTTCCTC-3' (forward for ΔZF), 5'-AGCGGCCGCTCAT CGAGCCTGGAGATCCTTGGC-3' (reverse for ΔZF), 5'-AGAATTCCGAAGGCCTTCAACTGCAAATAC-3' (forward for ZF) and 5'-AGCGGCCGCTCAGCGGGGACATCC-3' (reverse for ZF). Using pCMV/Myc-NICD as a template, RAM domain-deleted (ΔRAM), ANK domain-deleted (ΔANK), TAD domain-deleted (ΔTAD), OPA domain-deleted (ΔOPA) and PEST domain-deleted (ΔPEST) mutant constructs were developed by site-directed mutagenesis. All constructs were confirmed by sequencing. HA-tagged ubiquitin and human Jagged1 constructs were kindly provided by Dr C Chung and Dr L Nie, respectively. Flag-Snail WT and 2SA mutant constructs were obtained from Addgene, Inc (Cambridge, MA, USA).

### Retroviral expression vector and infection

Flag-Snail and Myc-NICD were cloned into pMSCV. To generate MSCV-expressing Flag-Snail or Myc-NICD, we transfected GP293 cells with pMSCV (for control virus), pMSCV/Flag-Snail or pMSCV/Myc-NICD with FuGENE 6 transfection reagent. Twenty-four hours after transfection the medium was changed, then the medium was collected at 12-hour intervals. The collected medium containing retrovirus was filtered and stored at -20°C. Before the retrovirus was used, we titrated it using the QIAamp Viral RNA Mini Kit (QIAGEN, Valencia, CA, USA) and the One Step SYBR PrimeScript RT-PCR Kit II (TaKaRa Bio Inc, Shiga, Japan). Cells were seeded at 30% confluence 12 hours before infection, and the media were replaced with medium containing MSCV. After infection for 24 hours, the medium was replaced with fresh medium and the infected cells were selected with puromycin (InvivoGen).

### Antibodies

In this study, we used the following antibodies: rat and mouse anti-Snail mAb (Cell Signaling Technology, Inc, Danvers, MA, USA), rabbit anti-Snail pAb (Abcam, Cambridge, MA, USA), rabbit anti-Notch1 mAb (Epitomics, Inc, Burlingame, CA, USA), goat anti-Notch1 pAb (Santa Cruz Biotechnology, Santa Cruz, CA, USA), rabbit anti-Jagged1 pAb (Santa Cruz Biotechnology), rabbit anti-p21 pAb (Cell Signaling Technology, Inc, and Santa Cruz Biotechnology), mouse anti-E-cadherin mAb (BD Biosciences, Franklin Lakes, NJ, USA), mouse anti-Myc mAb (Santa Cruz Biotechnology), mouse anti-Flag mAb (Sigma-Aldrich), mouse anti-HA mAb (Sigma-Aldrich), rabbit anti-HA pAb (Abcam) and mouse anti-β-actin mAb (Sigma-Aldrich).

### Immunoblot analysis, immunocytochemistry and immunoprecipitation

Tissue and cell lysates were prepared, and immunoblot analysis was performed as described previously [26]. Band intensity was determined using ImageMaster 2D Elite version 4.01 software (Amersham/GE Healthcare, Uppsala, Sweden). For immunoprecipitation, Hep3B cells were transfected with Flag-Snail, Myc-NICD and MDM2. After 48 hours, the cells were lysed in buffer (50 mM Tris·HCl, pH 8.0, 150 mM NaCl, 5 mM ethylenediaminetetraacetic acid (EDTA) and 0.5% Nonidet P-40 (NP-40)) and centrifuged at 16, 000 × *g *for 15 minutes to remove debris. Cleared lysates were subjected to immunoprecipitation with antibodies. For immunocytochemistry, cells were fixed in 4% paraformaldehyde at room temperature for 15 minutes, permeabilized in 5% Triton X-100 for 5 minutes, and then stained using pAbs. The secondary antibodies used were anti-mouse Alexa Fluor 594 dye conjugate and anti-rabbit Alexa Fluor 488 dye conjugate (Molecular Probes/Life Technologies, Carlsbad, CA, USA). Nuclei were stained with 4', 6-diamidino-2-phenylindole (DAPI blue) (Molecular Probes/Life Technologies). After mounting, the cells were visualized using a multiphoton confocal laser-scanning microscope (Carl Zeiss, Thornwood, NY, USA).

### In vivo ubiquitination assay

Twenty-four hours after transfection the cells were treated with 10 μM MG132 for 6 hours. The treated cells were then harvested with PBS containing 10 mM *N*-ethylmaleimide (NEM) and 1 mM dithiothreitol (DTT). The cells were washed with PBS, centrifuged and subjected to one freeze-thaw cycle. Cell pellets were then resuspended in 200 μl of buffer 1 (10 mM Tris·HCl, pH 7.5, 10 mM NaCl, 0.5% NP-40, 5 mM EDTA, 1 mM ethylene glycol tetraacetic acid (EGTA), 10 mM NEM, 1 mM DTT, 5 mM NaF, 1 mM Na_3_VO_4 _and protease inhibitor cocktail) and sonicated in a water bath (Bioruptor; Diagenode, Denville, NJ, USA). Next 500 μl of buffer 2 (20 mM Tris·HCl, pH 7.5, 0.5 M NaCl, 0.5% NP-40, 5 mM EDTA, 1 mM EGTA, 10 mM NEM, 1 mM DTT, 5 mM NaF, 1 mM Na_3_VO_4 _and protease inhibitor cocktail) were added, and the extracts were subjected to a 30-minute rotation at 4°C. Extracts were then centrifuged. We added 2 μg of anti-Flag M2 antibody and protein A/G beads to the supernatant and incubated it for 2 hours. The beads were then washed three times, resuspended in loading buffer and boiled. Immunoblotting was performed as described above.

### Real-time PCR analysis

Real-time PCR analysis of cDNA samples was performed with specific primers designed using Primer Express software (Applied Biosystems, Foster City, CA, USA). The primers used for Snail were 5'-AAGGATCTCCAGGCT CGAAAG-3' (forward) and 5'-GCTTCGGATGTGCATCTTGA-3' (reverse) and the primers used for β-actin were 5'-GCAAAGACCTGTACGCCAACA-3' (forward) and 5'-TGCATCCTGTCGGCAATG-3' (reverse). Total RNA was extracted from cultured cells using an RNeasy kit (QIAGEN) according to the manufacturer's protocol. cDNA was synthesized using 1 μg of RNA with avian myeloblastosis virus reverse transcriptase (Promega, Madison, WI, USA) and oligo(dT) primers. Transcript levels were assessed by quantitative real-time PCR (ABI 7300; Applied Biosystems), and all experiments were normalized to β-actin.

### Invasion assays

Cell invasion was measured at 96 hours as described by the manufacturer (Oris Cell Invasion and Detection Assay Kit; Platypus Technologies, LLC, Madison, WI, USA). In the invasion assay, fluorescence was monitored at an excitation of 492 nm and an emission of 530 nm using a multiplate reader (EnVision Multilabel Reader; PerkinElmer, Inc, Waltham, MA, USA).

### Duolink II fluorescence assay

The Duolink II fluorescence assay was used to analyze Hep3B, HT-29, Panc-1 and MDA-MB231 cells. Cells were seeded at 30% confluence on a cover glass and treated with 10 μM MG132 and 300 μM H_2_O_2 _for 6 hours. Huh7 cells were fixed with 4% paraformaldehyde and permeabilized with 0.5% Triton X-100 for 15 minutes each, then blocked and incubated with mouse anti-Snail Ab (1:100 dilution; Cell Signaling Technology) and rabbit anti-Notch1 Ab (1:100 dilution; Epitomics, Inc) for 30 minutes. The Duolink II PLA probe protocol was used to detect the signals. After mounting, the cells were visualized using a multiphoton confocal laser-scanning microscope (Carl Zeiss).

### Glutathione S-transferase **pull-down assay**

Bacterial lysate expressing GST-Snail proteins (WT, ΔZF and ZF mutants) was purified by immobilization on glutathione Sepharose beads (Pharmacia Biotech/GE Healthcare, Uppsala, Sweden). The beads were thoroughly washed with wash buffer (20 mM Tris·HCl, pH 8.0, 1 mM EDTA, 1 mM DTT, 150 mM NaCl, 1% Triton X-100) containing protease inhibitor mixture. The bound proteins were incubated with Myc-NICD-transfected cell lysate, washed with the wash buffer and eluted by boiling in the SDS sample buffer for 10 minutes. The sample was then analyzed by immunoblotting.

### Statistical analysis

The data in the bar graphs of Figure 3 and 6 are expressed as the means (± SD) of three independent experiments. All results shown in the bar graphs are expressed as the fold ratio relative to untreated or control cells. Statistical analysis was performed using SPSS version 12 statistical software (SPSS Inc, Chicago, IL, USA).

## Abbreviations

ANK: 6 ankyrin/cdc10 repeat; CSL: CBF1/RBPJk in vertebrates: Suppressor of hairless in *Drosophila*: Lag-1 in *Caenorhabditis elegans*; DMEM: Dulbecco's modified Eagle's medium; EMT: epithelial-to-mesenchymal transition; HCC: hepatocellular carcinoma; mAb: monoclonal antibody; MEF: mouse embryonic fibroblast; MS: mass spectrometry; MSCV: murine stem cell virus; MS/MS: tandem affinity purification and mass spectrometry; Mut: mutant; NICD: Notch1 intracellular domain; OPA: polyglutamine tract; pAb: polyclonal antibody; PBS: phosphate-buffered saline; PCR: polymerase chain reaction; PEST: proline-: glutamic acid-: serine- and threonine-rich region; ROS: reactive oxygen species; RT-PCR: reverse transcriptase polymerase chain reaction; siRNA: small interfering RNA; SNAG: divergent N-terminal region; TAD: transcriptional transactivation domain; WT: wild type; ZF: zinc finger.

## Competing interests

The authors declare that they have no competing interests.

## Authors' contributions

SOL carried out the majority of the experiments in this study, designed study, and wrote manuscript. HSK carried out protein interaction and siRNA experiments and edited manuscript. XQ carried out siRNA experiments. SMA carried out the interaction domain screen. HK participated in study design and gave critical discussions. DH edited manuscript. JKS provided mice for MEF, GJ designed study and edited manuscript. All authors read and approved the final manuscript.

## Supplementary Material

Additional file 1 **Peptides sequence of identified Snail-bound proteins including Snail by mass spectrometry analysis**. Click here for file

Additional file 2 **Notch1 interacts with Snail**. HT-29, Panc-1, or MDA-MB-231 cells were immunostained with anti-Snail and/or anti-Notch1 antibodies and assessed by the Duolink^® ^II assay. Red spots indicate the interaction between the endogenous Snail and Notch1 proteins. Click here for file

Additional file 3 **Snail and NICD regulate invasion**. (A-C) MEFs (A), Huh7 (B), and Hep3B (C) were infected by MSCV-NICD and/or MSCV-Snail, selected in puromycin, and analyzed for Notch1 and Snail expression by immunoblot with the indicated antibodies. β-actin served as an internal control. (D) Hep3B cells were transfected by Notch1 and/or Snail siRNA, treated with 300 μM H_2_O_2 _for 72 h, and analyzed for Notch1 and Snail expression by immunoblot with the indicated antibodies. E-cadherin, which is a Snail target gene, served as a marker of Snail activity. β-actin served as an internal control. (E) Hep3B cells were transfected by Notch1 and/or Snail siRNA, treated with 300 μM H_2_O_2 _for 72 h, and analyzed for Notch1 and Snail expression by immunoblot with the indicated antibodies. β-actin served as an internal control. Click here for file
