# Supplementary material for: Notch1 binds and induces degradation of Snail in hepatocellular carcinoma
Source: BMC Biol. 2011 Nov 30;9:83. doi: 10.1186/1741-7007-9-83 (PMC3247845; doi:10.1186/1741-7007-9-83)
Supplement: Additional file 1 — Peptides sequence of identified Snail-bound proteins including Snail by mass spectrometry analysis. [file 1741-7007-9-83-S1.DOC]

**Additional file 1. Peptides sequence of identified Snail-bound proteins including Snail by mass spectrometry analysis.**

| **Protein name** | **Accession number** | **Sequence** |
| --- | --- | --- |
| Snail | 12644089 | VAELTSLSDEDSGK  QLAQLSEAK  DLQAR  EYLSGALK  AFSRPWLLQGHVR |
| HSP70 | 55962553 | VEIIANDQGNR  TTPSYVAFTDTER  HWPFQVINDGDKPK  VQVSYKGETK  AFYPEEISSMVLTK  DAGVIAGLNVLR |
| Importin 8 | 45477008 | IINFAPSLLR  VLQQAFNYLNQGVVHSITWK  TALQEVYTLAEHR |
