# Supplementary material for: Notch1 binds and induces degradation of Snail in hepatocellular carcinoma
Source: BMC Biol. 2011 Nov 30;9:83. doi: 10.1186/1741-7007-9-83 (PMC3247845; doi:10.1186/1741-7007-9-83)
Supplement: Additional file 2 — Notch1 interacts with Snail. HT-29, Panc-1, or MDA-MB-231 cells were immunostained with anti-Snail and/or anti-Notch1 antibodies and assessed by the Duolink® II assay. Red spots indicate the interaction between the endogenous Snail and Notch1 proteins. [file 1741-7007-9-83-S2.DOC]

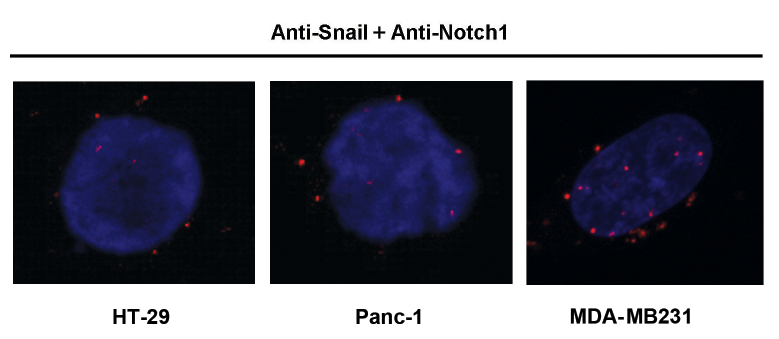


**Additional file 2. Notch1 interacts with Snail.** HT-29, Panc-1, or MDA-MB-231 cells were immunostained with anti-Snail and/or anti-Notch1 antibodies and assessed by the Duolink® II assay. Red spots indicate the interaction between the endogenous Snail and Notch1 proteins.
