# Supplementary material for: Notch1 binds and induces degradation of Snail in hepatocellular carcinoma
Source: BMC Biol. 2011 Nov 30;9:83. doi: 10.1186/1741-7007-9-83 (PMC3247845; doi:10.1186/1741-7007-9-83)
Supplement: Additional file 3 — Snail and NICD regulate invasion. (A-C) MEFs (A), Huh7 (B), and Hep3B (C) were infected by MSCV-NICD and/or MSCV-Snail, selected in puromycin, and analyzed for Notch1 and Snail expression by immunoblot with the indicated antibodies. β-actin served as an internal control. (D) Hep3B cells were transfected by Notch1 and/or Snail siRNA, treated with 300 μM H2O2 for 72 h, and analyzed for Notch1 and Snail expression by immunoblot with the indicated antibodies. E-cadherin, which is a Snail target gene, served as a marker of Snail activity. β-actin served as an internal control. (E) Hep3B cells were transfected by Notch1 and/or Snail siRNA, treated with 300 μM H2O2 for 72 h, and analyzed for Notch1 and Snail expression by immunoblot with the indicated antibodies. β-actin served as an internal control. [file 1741-7007-9-83-S3.DOC]

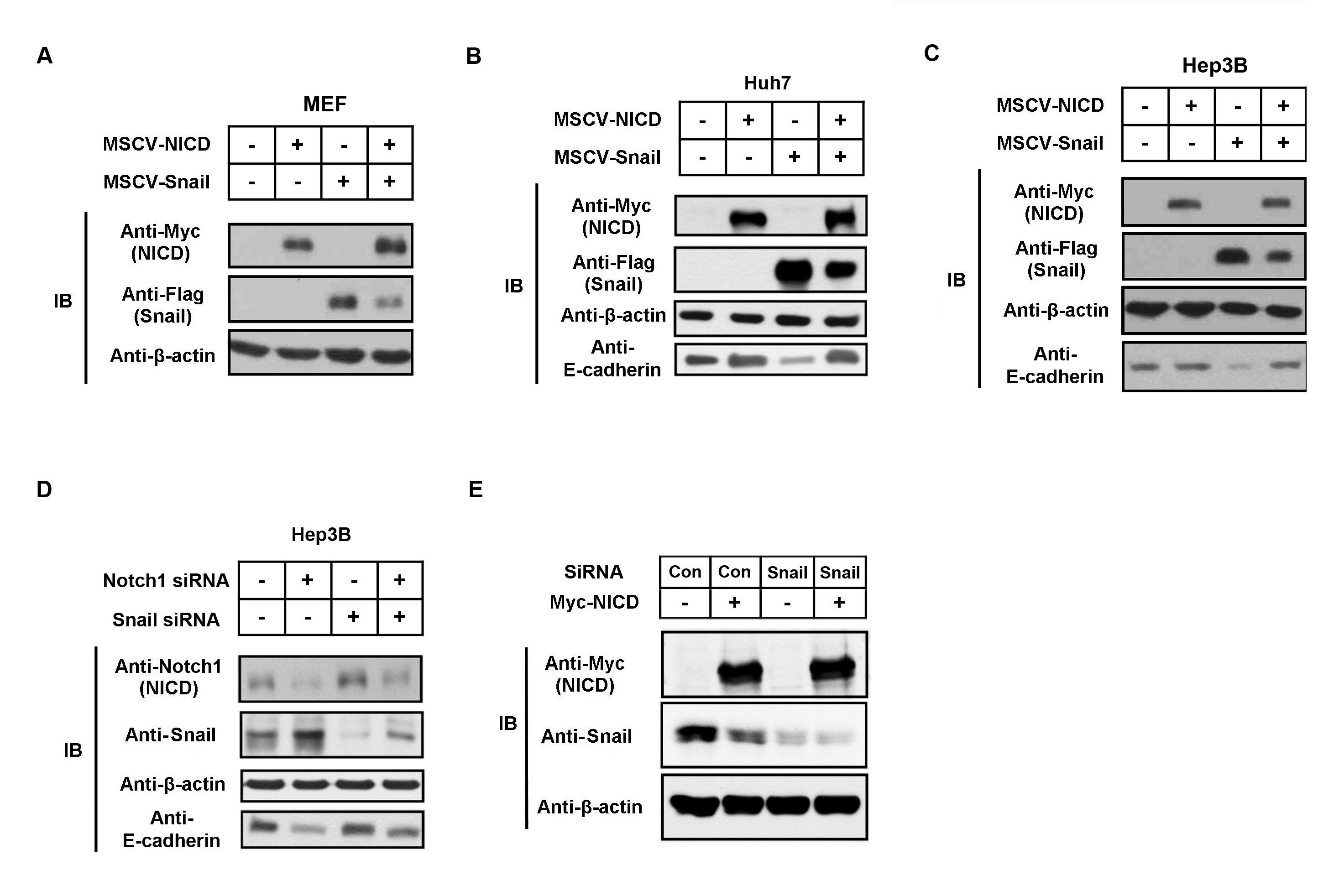


**Additional file 3. Snail and NICD regulate invasion.** (A-C) MEFs (A), Huh7 (B), and Hep3B (C) were infected by MSCV-NICD and/or MSCV-Snail, selected in puromycin, and analyzed for Notch1 and Snail expression by immunoblot with the indicated antibodies. β-actin served as an internal control. (D) Hep3B cells were transfected by Notch1 and/or Snail siRNA, treated with 300 µM H_2_O_2_ for 72 h, and analyzed for Notch1 and Snail expression by immunoblot with the indicated antibodies. E-cadherin, which is a Snail target gene, served as a marker of Snail activity. β-actin served as an internal control. (E) Hep3B cells were transfected by Notch1 and/or Snail siRNA, treated with 300 µM H_2_O_2_ for 72 h, and analyzed for Notch1 and Snail expression by immunoblot with the indicated antibodies. β-actin served as an internal control.
